# Microseeding – A Powerful Tool for Crystallizing Proteins Complexed with Hydrolyzable Substrates

**DOI:** 10.3390/ijms9071131

**Published:** 2008-07-08

**Authors:** Christine Oswald, Sander H. J. Smits, Erhard Bremer, Lutz Schmitt

**Affiliations:** 1 Institute of Biochemistry, Heinrich Heine University Duesseldorf, Universitaetsstrasse 1, 40225 Duesseldorf, Germany; 2 Laboratory for Microbiology, Department of Biology, Philipps University Marbrug, Karl-von-Frisch Str., 35032 Marburg, Germany

**Keywords:** crystal twinning, acetylchline binding protein, co-crystallization

## Abstract

Hydrolysis is an often-encountered obstacle in the crystallization of proteins complexed with their substrates. As the duration of the crystallization process, from nucleation to the growth of the crystal to its final size, commonly requires several weeks, non-enzymatic hydrolysis of an “unstable” ligand occurs frequently. In cases where the crystallization conditions exhibit non neutral pH values this hydrolysis phenomenon may be even more pronounced. ChoX, the substrate binding protein of a choline ABC-importer, produced crystals with its substrate acetylcholine after one month. However, these crystals exhibited only choline, an acetylcholine hydrolysis product, in the binding site. To overcome this obstacle we devised a microseeding protocol leading to crystals of ChoX with bound acetylcholine within 24 hours. One drawback we encountered was the high twinning fraction of the crystals, possibly was due to the rapid crystal growth.

## 1. Introduction

The process of obtaining structural information by protein X-ray crystallography has witnessed great advances in the last decades. However, obtaining crystals suitable for data collection still represents the bottleneck in structure determination. Unfortunately, the crystallization of proteins still is a trial and error process that can be very time consuming. Often crystals need weeks until they appear or grow to a size which is useful for crystal mounting and subsequently data collection. During this period, the protein can suffer degradation leading to incomplete protein structures. In cases where the aim is a co-crystallization with a substrate, not only protein degradation but also chemical changes of the substrates can present an obstacle, for example, if the substrate is converted by an active enzyme or if it is unstable in solution over the time course of a classical crystallization experiment. Therefore, to obtain a structure inactive mutants of the enzyme are often used complexed with the selected substrate [1, 2]. The application of an inert (i.e. non-cleavable or non-hydrolyzable) mimic of the substrate in crystallization presents another possibility to obtain an enzyme/substrate structure. However, sometimes these mimics are not able to fully resemble the native substrate as it was shown for the ATP mimic AMP-PNP in case of ABC transporters [3].

ABC transporters [4] are found in all phyla of life [5]. These transmembrane proteins utilize the energy derived by ATP binding and/or hydrolysis to energize the vectorial transport of a substrate across cellular membranes. Thereby, transporters can function as importers or exporters. In contrast to exporters, which consist of a minimal core of two nucleotide-binding domains and two transmembrane domains, importers exhibit an additional domain, the substrate binding protein (SBP). In Gram-negative bacteria this domain is located in the periplasmic space, whereas in Gram-positive bacteria and archaea it is anchored to the membrane via a lipid modification or by a fusion to transmembrane peptide [6]. The function of substrate binding proteins is to capture the substrate and deliver it to the transporter. To efficiently accomplish this task the SBP is equipped with a high specificity and affinity for its substrate [7].

*Sinorhizobium meliloti* (*S. meliloti*) is a Gram-negative bacterium that can be found in soil and the rhizosphere. *S. meliloti* lives in symbiosis with certain plants and induces the formation of nodules at the root of the plants. Choline, a compound readily found in the rhizosphere, plays a major role for *S. meliloti*. It can be used as phosphatidylcholine precursor and, after conversion to glycine betaine, as carbon and nitrogen source or as osmoprotectant. Dependent on the usage of choline different transport systems have developed in *S. meliloti*. One of these choline transporters, the ChoVWX, belongs to the superfamily of ABC transports [8]. Its substrate binding protein, ChoX, shows a high affinity for its natural substrate choline. Additionally, *in vitro* studies on ChoX revealed a medium affinity for acetylcholine [8]. To understand the binding properties of ChoX, a structural determination of ChoX in complex with acetylcholine was aimed for. For this purpose the protein was subjected to co-crystallization experiments with acetylcholine. However, subsequent structural determination showed that the substrate was hydrolyzed to choline in the setup during the time of crystal growth.

To overcome this limitation, a microseeding strategy was devised. Here, we discuss the advantages of microseeding as a method to induce rapid crystal formation thereby circumventing the hydrolysis of acetylcholine during the crystallization process. The application of microseeding enabled us to crystallize ChoX complexed with acetylcholine in under 24 hours. Structural analysis revealed that acetylcholine was not hydrolyzed in the drop during this short crystal growth time. On the other hand, crystals produced by this strategy were all perfect pseudo-merohedral twins. Therefore, we also discuss the relation between seeding, twinning and the time required for crystal formation.

## 2. Methods

### 2.1. Expression and purification of ChoX

The expression and purification of ChoX was performed as described elsewhere [8]. Briefly, ChoX was expressed using a pET20b vector in *E. coli* BL21 (DE3) (pLysS). Cells were grown in minimal media and thereafter subjected to periplasmic cell rupture by an osmotic shock. To remove cell debris and unbroken cells the suspension was treated by ultracentrifugation. The obtained supernatant was purified via affinity purification through a C-terminal hexahistidine tag. The protein was eluted by an imidazole gradient (10 to 250 mM). Buffer exchange for crystallization experiments was accomplished by dialysis against 10 mM Tris/HCl pH 7.0.

### 2.2. Crystallization of ChoX/acetylcholine

Crystals of ChoX as well as the mutant of ChoX complexed with acetylcholine were grown in a vapor diffusion hanging drop setup using the streak-seeding method. The precipitant solutions used in the microseeding setups contained 100 mM sodium acetate pH 5.0 and 16% PEG 3350. Protein and precipitant were mixed in a 1:1 ratio to a final drop volume of 2 μL. Additionally, the drops were supplemented with acetylcholine to a final concentration of 1 mM. With the help of a horse tail hair, several micro-crystals from a ChoX/choline crystal were transferred into the new crystallization setup. The plates were sealed and subsequently stored at 4 °C. After 24 hours crystals, which were already large enough to be used for data collection (100 × 50 × 20μm), were mounted in a cryo loop and directly frozen in liquid nitrogen.

### 2.3. Data collection, structure refinement and analysis

Data processing and structure calculation are described elsewhere (Oswald *et al*., submitted for publication) but shall be described here briefly. Data sets were collected at beamline BW7A of the EMBL outstation at DESY Hamburg. The dataset was processed with the XDS program package [9] and subsequently subjected to twinning analysis utilizing the algorithm of Padilla and Yeates [10], which revealed an almost perfect twin. Crystals belonged to space group P21 with cell axis of a= 31.2 Å, b= 212.7 Å and c= 42.8 Å (β = 90.1°) with two protomers in the asymmetric unit. Molecular replacement was performed utilizing the program MOLREP [11]. As search model, the structure of ChoX/choline (PDB code: 2REG) was depleted of its ligands and water molecules and further reduced to one monomer of ChoX. Further refinement consisted of iterative rounds of restrained refinement, carried out with SHELXL97–2 [12] using (h,-k,-l) as twinning operator, and manual rebuilding using COOT [13]. Initially, water molecules were detected using ARP/wARP [14] (threshold of 3.2 sigma) and thereafter their position was manually checked. The model was refined to a R_F_ value of 15.1 and a R_free_ value of 21% at a resolution of 1.8 Å. Figures were generated using PyMol [15].

## 3. Results and Discussion

### 3.1. Acetylcholine is hydrolyzed in vapor diffusion crystallization set-ups

*In vitro* analysis of the binding characteristics of ChoX revealed a high affinity for choline (K_d_ 2.3 ± 1.0 μM) (Oswald *et al*; submitted for publication). Surprisingly, not only the natural transport substrate choline is able to interact with the binding protein [8]. Acetylcholine can also bind to ChoX with moderate affinity (K_d_ 100 ± 12 μM) (Oswald *et al*; in preparation).

In order to reveal the binding properties of both ligands, ChoX was subjected to structural analysis. For this purpose ChoX was crystallized in a conventional vapor diffusion setup supplemented with an excess of the respective ligand. Independent of the supplemented ligand, protein crystals appeared within 4 weeks. However, in the case of acetylcholine structural analysis revealed only little electron density in the binding site. In fact the electron density obtained from the crystal grown in an acetylcholine supplemented setup more resembled the density obtained from the co-crystallization experiment of ChoX with choline. This finding suggested that the ligand situated in the binding site indeed is choline, obtained after the hydrolysis of acetylcholine. The crystals which were subjected to analysis were grown from a precipitant solution containing acetate buffer at a pH around 4.5. Furthermore, the crystals only appeared within one month. Therefore, it seems very likely that acetylcholine was hydrolyzed during the time the crystals needed to emerge. This assumption is supported by biochemical and structural work on acetylcholine esterase and the acetylcholine binding protein [16]. The hydrolysis process is probably catalyzed by the acidic environment of the setup. The products of this hydrolysis, acetate and choline, further compete with acetylcholine for the binding site. As choline exhibits a higher affinity to ChoX than acetylcholine, the prevailing ChoX species in the setup will hold choline in the same binding site. This explains why the obtained crystals consisted of ChoX/choline complexes instead of ChoX/acetylcholine ones. Nevertheless, the acetate is diffused away in the crystallization drop and not found inside the crystal, suggesting that hydrolysis occurs before the initial crystallization nuclei is formed, although we can not completely rule out that hydrolysis can also occur within the crystals.

**Figure 1.**
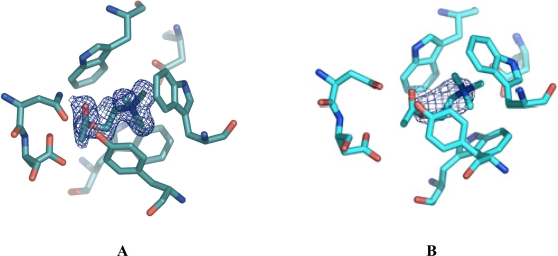
2F_o–_F_c_ electron density maps around the ligand-binding site of ChoX countered at 1 sigma. The density was calculated in the absence of a substrate. The displayed acetylcholine was placed manually in the corresponding density afterwards. For clarity, residues forming the ligand binding-site are shown in ball-and-sticks representation. A) Electron density (blue) derived from a crystal grown in a conventional vapor diffusion setup. The resolution of the data set was 1.9 Å. The size of the density is too small to cover the acetylcholine molecule. Acetylcholine was possibly hydrolyzed in the setup. B) Electron density (blue) derived from a crystal grown with the help of microseeding at a resolution of 1.8 Å. The electron density covers the whole acetylcholine molecule.

Figure 1A displays a final 2F_o–_F_c_ electron density map (colored blue) contoured at 1 sigma, of the ligand in the ChoX/acetylcholine setup. For clarity, amino acid residues participating in ligand binding are shown. Here, the density was obtained from a crystal of a conventional vapor diffusion setup with acetylcholine at a resolution of 1.9 Å. However, the present density does not cover the entire acetylcholine ligand. Rather, the density strongly supported our notion that the acetylcholine was hydrolyzed during the time of crystal formation and only choline was bound to the protein. However, we cannot completely rule out the presence of a mixture of choline and acetylcholine in the crystals obtained by the conventional co-crystallization set-up. If this were indeed the case, these crystals would be unsuitable to determine the ChoX/acetylcholine structure.

### 3.2. Seeding for quick crystal growth

Co-crystallization of ChoX with acetylcholine in a conventional vapor diffusion setup was unsuccessful as the ligand was hydrolyzed during the time the crystal needed to grow. To circumvent this problem we anticipated that either crystallization of ChoX/acetylcholine at neutral pH values or accelerated crystal growth was required. Although different alternative crystallization conditions were tested, none of them produced suitable crystals of ChoX complexed with acetylcholine. In order to obtain crystals containing fully preserved acetylcholine acceleration of the crystallization process seemed crucial.

The microseeding method promised to be most successful for this purpose. This technique takes advantage of the fact that the formation of a crystal is a two step process divided into nuclei formation and crystal growth. The initial step, nuclei formation, is more likely to occur if the protein solution is highly supersaturated [17]. In contrast, the growth of crystals, an ordered process, is maintained in the metastable zone of the phase diagram. Seeding methods separate the two events of nucleation and crystal growth [18]. In microseeding this separation is accomplished by transferring a seed, a submicroscopic crystal, from one condition, where the level of supersaturation is high, to a similar condition at a lower level of supersaturation. In order to have lower levels of supersaturation either the protein or the precipitant concentration is lowered in a crystallization setup [19].

**Figure 2.**
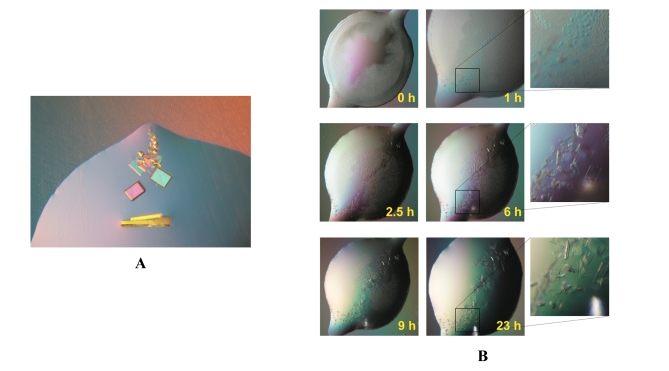
Pictures of crystals grown with the help of microseeding. A) The crystals grow along the streak seeding line. B) Time course of a seeding experiment. Crystals suitable for data collection are obtained in less than 24 hours.

As no crystals of ChoX complexed with acetylcholine were available a crystal of ChoX with its natural ligand choline was utilized to obtain seeds. Submicroscopic seeds, obtained by touching the crystal with a horse tail hair [18], where transferred by streak-seeding [20] to a freshly setup drop supplemented with acetylcholine instead of choline. As the crystallization of ChoX complexed with acetylcholine required rapid crystal growth, the seeding conditions were chosen in such a way that high saturation would be guaranteed. Therefore, the precipitant concentration was not lowered in comparison to conditions in which ChoX/choline crystals were obtained. Crystals of ChoX complexed with its new ligand grew along the streak seeding line, as exemplified in Figure 2A. By utilizing the microseeding method it was possible to obtain initial, small crystals of ChoX with acetylcholine in less than 3 hours. These crystals grew to sizes suitable for data collection within 24 hours. After 24 hours these crystals were harvested and flash frozen in liquid nitrogen to prevent further hydrolysis of acetylcholine. A time course of the crystal growth process utilizing the microseeding method is depicted in Figure 2B.

Astonishingly, the quality of these crystals was extremely high, showing diffraction up to 1.8 Å. The resulting electron density in the binding site is well defined for the acetylcholine molecule. Figure 1B shows the electron density, colored blue, in the binding site after molecular replacement and one round of restrained refinement in Refmac5. Here, the same search model as in Figure 1A was utilized for molecular replacement, meaning that no ligand was present in the binding site. Figure 1A only shows little density, which is probably accounting for a partial occupation of the binding site with a choline molecule. In contrast, the electron density in Figure 1B is much larger and of the defined shape of an acetylcholine molecule.

### 3.3. Is seeding inducing twinning?

**Figure 3.**
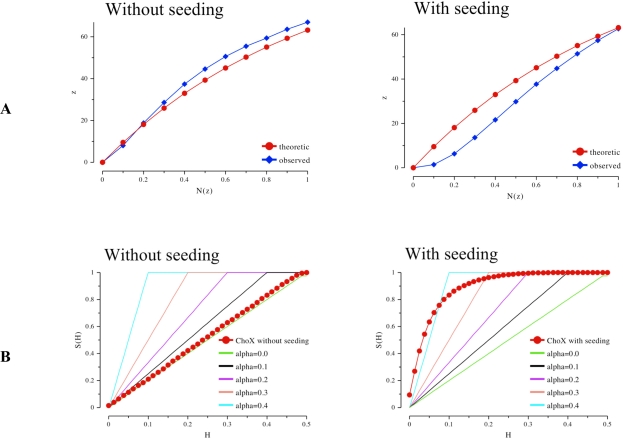

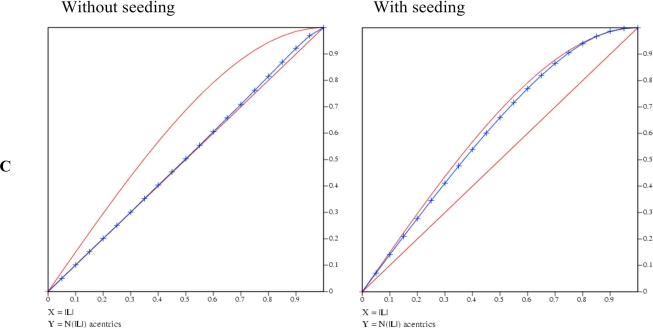
A) The Rees Plot of intensities. Left: intensity statistics derived from the collected dataset of ChoX crystals grown in a conventional vapor diffusion setup supplemented with acetylcholine. The intensities follow the known statistics for an untwinned crystal. Right: statistics of a highly twinned ChoX/acetylcholine crystal grown utilizing the microseeding method. B) The Yeates Plot of intensities. Left: intensity statistics derived from the collected dataset of ChoX crystals grown in a conventional vapor diffusion setup supplemented with acetylcholine. The intensities follow the common statistics for an untwinned crystal. Right: Statistics of a highly twinned ChoX/acetylcholine crystal grown utilizing the microseeding method. C) The L-function of intensities. Left: intensity statistics derived from the diffraction of ChoX crystals grown in a conventional vapor diffusion setup supplemented with acetylcholine. The intensities follow the common statistics for a non-twinned crystal. Right: the dataset statistics of a twinned ChoX/acetylcholine crystal grown with the help of the microseeding method.

To avoid hydrolysis during the crystallization process ChoX was crystallized complexed with acetylcholine utilizing a microseeding setup. Establishing microseeding resulted in very rapid crystal growth (less than 24 hours) of ChoX harboring the non-hydrolyzed substrate. Crystals of ChoX complexed with choline all exhibited space group P21. However, the unit cell parameters of the crystals obtained by microseeding experiments showed a β angle near 90°. Furthermore, the data set scaled equally well in an orthorhombic lattice, which was the first warning sign of twinning. In a monoclinic space group, twinning is rather uncommon, but might occur if the β angle is close to 90°, as it was the case in our study. Therefore, the data was carefully subjected to twinning analysis. Figure 3 shows different statistical data analysis of reflection data derived from a crystal grown in a conventional vapor diffusion setup (without seeding) and a crystal obtained by microseeding (with seeding).

The Rees plots [21] (Figure 3A) of both datasets give a first indication of crystal twinning. Here, the fraction of local average intensity of the acentric reflections z is plotted against the cumulative distribution of z. The red curve shows the theoretic contribution in case of non-twinned data, which follows an exponential progression. However, the crystal obtained by seeding shows a lower amount of weak intensities. The more sigmoidal progression is another indication for twinning [21]. Analysis according to Yeates not only indicates twinning in case of the crystal obtained by seeding but it also provides a first estimate for the twinning factor α [22, 23].

Whereas the crystal grown by conventional vapor diffusion setup shows no twinning the crystal from the microseeding experiment shows a high twinning factor above 0.4 (Figure 3B). A very robust test for twinning which also gives a good estimate for the twinning factor and is rather insensitive to anisotropic diffraction is an analysis using the L-function [10] (blue curve in Figure 3C). The analysis of the twinned crystal shows an extremely high value for α approaching almost 0.5, the value for a perfect twin (upper red curve). In contrast the crystal of the conventional vapor diffusion setup shows no twinning as its L-function follows the progression for untwinned data (lower red curve).

With the help of microseeding it was possible to acquire crystals within a few hours. However, these crystals showed a high twinning fraction in contrast to the crystals grown under conventional conditions. Therefore, the question arises wether there is a correlation between seeding and twinning. In a conventional setup, where nuclei need to be formed first, the nucleation process is guided by the nucleation barrier [24]. In contrast, when a seeding experiment is conducted and nuclei are provided this nucleation barrier is of minor importance. Thus, a seeding experiment requires a lower saturation level than a conventional crystallization setup [18, 20].

However, in order to drastically accelerate the growth process for ChoX with acetylcholine conditions supporting a very high level of saturation were chosen. Thereby, the equilibrium between the segregation of a solid phase (crystal) and the dissolving of the newly formed phase is also influenced. Thus, the new solid phase forms very rapidly. Unfortunately, this enhances the occurrence of crystal growth disorders, such as twinning, as possible defects in crystal development are less likely to be dissolved. However, this also means that crystals grown less rapidly possibly exhibit a lower degree of twinning.

This hypothesis was tested with a mutant crystal of ChoX. Here the crystal appeared 3 days after streak seeding was used to induce crystal formation and indeed showed a lower twinning fraction (α∼ 0.35). This supported our hypothesis that the twinning fraction in the case of the ChoX/acetylcholine crystals was indeed dependent on the time of crystal grows. However, one has to keep in mind that twinning was only possible due to a beta angle close to 90°. Thus, the strategy outlined here, demonstrates the power of microseeding to obtain X-ray suitable crystals of labile substrates. An analysis of twinning is easily performed nowadays and even in our case (perfect pseudo-merohedral twinning), the crystal structure could successfully be solved (Oswald *et al.* submitted for publication).

## 4. Conclusions

Although structure determination of proteins by X-ray crystallography has received a great boostduring the last decades, there are still some obstacles which may delay or even hinder a successful structure analysis of certain species. For example, in cases where enzymes are studied that are capable of converting the substrate to the product during the growth phase of the crystals, problems in crystal formations or, as shown here, selection of the ligand inside the binding pocket might occur. In this case, often mutants deficient in activity, are the best choice for a structural analysis [1, 2]. Another possibility is to use substrate mimics like for example the ATP analogs AMPPNP or ATP-γ-S, which are only slowly or not at all converted. However, sometimes these mimics cannot represent the natural substrate in all aspects.

Acetylcholine is a chemical compound which is easily susceptible to hydrolysis, especially at non-neutral pH values. As the crystallization of ChoX is conducted at low pH values, a co-crystallization with intact acetylcholine could not be achieved. Nevertheless, establishing microseeding enabled the crystallization of the ligand bound species within less than 24 hours, which greatly reduced a modification of the substrate due to hydrolysis. Thereby, it was possible to solve the structure of ChoX in complex with acetylcholine. Surprisingly, the quality of the crystals was extremely good, resulting in diffraction up to 1.8 Å. However, one drawback encountered, when crystals of ChoX, were obtained by seeding was that they all showed a high twinning fraction (up to 50%). This effect is possibly due to the rapid growth process where crystals reach their final size within a day allowing the formation of pseudo-merohedral twins.

By carefully choosing the degree of saturation, i.e. varying precipitant or protein concentration, it is possible to balance the minimization of twinning and the substrate decay, in this case hydrolysis. The great advantage of low twinning fractions lies within the possibility to apply a detwinning procedure on the reflection intensities [10, 21, 23]. Thereafter, the structure calculation can be performed with the detwinned dataset. In contrast high twinning fractions necessitate the use of programs that can handle twinned data such as CNS [25], Phenix (www.phenix-online.org) or SHELXL [12]. This study highlights application of microseeding as a powerful tool to enable the crystallization of proteins with unstable substrates.

## References

[1] Zaitseva J, Jenewein S, Jumpertz T, Holland IB, Schmitt L (2005). H662 is the Linchpin of ATP Hydrolysis in the Nucleotide-binding Domain of the ABC Transporter HlyB. EMBO J.

[2] Bourne Y, Radic Z, Sulzenbacher G, Kim E, Taylor P, Marchot P (2006). Substrate and Product Trafficking through the Active Center Gorge of Acetylcholinesterase Analyzed by Crystallography and Equilibrium Binding. J Biol Chem.

[3] Verdon G, Albers SV, Dijkstra BW, Driessen AJ, Thunnissen AM (2003). Crystal Structures of the ATPase Subunit of the Glucose ABC Transporter from *Sulfolobus solfataricus*: Nucleotide-free and Nucleotide-bound Conformations. J Mol Biol.

[4] Linton KJ, Higgins CF (1998). The *Escherichia coli* ATP-binding Cassette (ABC) Proteins. Mol Microbiol.

[5] Higgins CF (1992). ABC Transporters: from Microorganisms to Man. Annu Rev Cell Biol.

[6] Biemans-Oldehinkel E, Doeven MK, Poolman B (2006). ABC Transporter Architecture and Regulatory Roles of Accessory Domains. FEBS Lett.

[7] Wilkinson J, Verschueren KHG (2003). ABC Proteins: From Bacteria to Man.

[8] Dupont L, Garcia I, Poggi MC, Alloing G, Mandon K, Le Rudulier D (2004). The *Sinorhizobium meliloti* ABC Transporter Cho is Highly Specific for Choline and Expressed in Bacteroids from Medicago Sativa Nodules. J Bacteriol.

[9] Kabsch W (1993). Automatic Processing of Rotation Diffraction Data from Crystals of Initially Unknown Symmetry and Cell Constants. J Appl Crystallogr.

[10] Padilla JE, Yeates TO (2003). A Statistic for Local Intensity Differences: Robustness to Anisotropy and Pseudo-centering and Utility for Detecting Twinning. Acta Crystallogr D.

[11] Vagin A, Teplyakov A (2000). An Approach to Multi-copy Search in Molecular Replacement. Acta Crystallogr D.

[12] Sheldrick GM, Schneider TR (1997). SHELXL: High-resolution Refinement. Meth Enzymol.

[13] Emsley P, Cowtan K (2004). Coot: Model-building Tools for Molecular Graphics. Acta Crystallogr D.

[14] Lamzin VS, Wilson KS (1993). Automated Refinement of Protein Models. Acta Crystallogr D.

[15] DeLano WL (2002). The PyMol Molecular Graphics System. http://www.pymol.org.

[16] Pandey PC, Upadhyay S, Pathak HC, Pandey CMD, Tiwari I (2000). Acetylthiocholine/Acetylcholine and Thiocholine/Choline Electrochemical Biosensors/Sensors Based on an Organically Modified Sol–Gel Glass Enzyme Reactor and Graphite Paste Electrode. Sens Actuat B.

[17] McPherson A (1999). Crystallization of Biological Macromolecules.

[18] Bergfors T (2003). Seeds to Crystals. J Struct Biol.

[19] van Aalten DMF, Milne KG, Zou JY, Kleywegt GJ, Bergfors T, Ferguson MAJ, Knudsen J, Jones TA (2001). Binding Site Differences Revealed by Crystal Structures of *Plasmodium falciparum* and Bovine Acyl-Coa Binding Protein. J Mol Biol.

[20] Stura EA, Wilson IA (1991). Applications of the Streak Seeding Technique in Protein Crystallization. J Cryst Growth.

[21] Rees DC (1980). The Influence of Twinning by Merohedry on Intensity Statistics. Acta Crystallogr A.

[22] Yeates TO (1988). Simple Statistics for Intensity Data from Twinned Specimens. Acta Crystallogr A.

[23] Yeates TO (1997). Detecting and Overcoming Crystal Twinning. Meth Enzymol.

[24] Manuel Garcia-Ruiz J (2003). Nucleation of Protein Crystals. J Struct Biol.

[25] Brünger AT, Warren GL (1998). Crystallography & NMR System: a New Software Suite for Macromolecular Structure Determination. Acta Crystallogr D.

